# Case of Extra Uterine Pregnancy

**Published:** 1846-01

**Authors:** William H. Mason

**Affiliations:** Tuckerton, N. J.


*Case of Extra Uterine Pregnancy.* By Dr. William H. Mason, of Tuckerton, N. J. [Communicated in a letter to Professor Pancoast.]

In April, 1841, I was called to A. P., who had borne several children, and was then, as she believed, in the fifth month of pregnancy. She had already felt the child sensibly, and expected to be confined in August following.

I visited her frequently from the time when I was first called, until the last of the following September. The latter part of August, she was seized with frequent, but irregular pains in the abdomen, which was now very much enlarged, and the motions of the fœtus so strong, as to cause her sometimes to scream out. In a few days the pains subsided, and the motions of the fœtus ceased, and it was therefore concluded that the child was dead. She now complained of great pain in the left hypochondriac region. On making an examination per vaginam, I found the os uteri but slightly dilated; the fœtal head high up, with a soft and heavy mass intervening. Could discover no motion of the fœtus. Having to leave home, my visits were now discontinued for some time.

Dec. 3. I was again called, and found her much emaciated, with an abscess in the part where she had complained of so much pain, (left hypochondriac region.) The size of the abdomen had been greatly reduced, about four weeks before, by an abundant discharge of fœtid water from the intestines. On passing a probe into the abscess it came in contact with bones. The abscess had been discharging externally for the last two weeks, and the lower extremities had become quite anasarcous. I visited her frequently from this date until her death, and at sundry times extracted portions of a dead fœtus, through the opening in the abscess, in a highly putrid condition, until I got all but the bones of the head, part of the arms, and some few of the smaller bones. About two weeks before her death, she had a hectic cough, and a copious expectoration of yellowish pus, but without much excitement of pulse, or general febrile action. Her appetite was generally good, until within two weeks of her death, when it failed, and she sunk rapidly and expired on the 25th of December.

*Necroscopy.*—In opening the abdomen, immediately under the umbilicus, came in contact with the parietal bones of the fœtus, contained in a thin membranous sac, not thicker than writing paper. The sac was firmly adherent to the peritoneum anteriorly, and posteriorly to the omentum. Under the umbilicus, in the portion of the sac adjoining the intestines, there was an opening of the size of a twenty-five cent piece, communicating with the intestine through which, in all probability, the fœtid discharge occurred, at the time of the sudden abatement of the abdominal swelling. A communication existed between the sac and the external opening in the side, but none with the vagina or uterus. The latter organ looked healthy, without any signs of having been ruptured, and was about the ordinary size of the unimpregnated uterus, the os uteri being normally closed. About a pint of very offensive matter was remaining in the sac.

